# Editorial: Harnessing the sustainable valorization and exploitation of salt tolerant plants

**DOI:** 10.3389/fpls.2023.1132119

**Published:** 2023-01-18

**Authors:** Luísa Custódio, Antonella Castagna, José A. Hernández, Christian Magné, Karim Ben Hamed

**Affiliations:** ^1^ Centre of Marine Sciences (CCMAR), University of Algarve, Campus de Gambelas, Faro, Portugal; ^2^ Department of Agriculture, Food and Environment, University of Pisa, Pisa, Italy; ^3^ Group of Fruit Trees Biotechnology, Centro de Edafología y Biología Aplicada del Segura-Consejo Superior de Investigaciones Científicas (CEBAS-CSIC), Murcia, Spain; ^4^ Géoarchitecture Territoires, Urbanisation, Biodiversité, Environnement, Faculty of Sciences and Techniques, Université de Bretagne Occidentale, Brest, France; ^5^ Laboratory of Extremophile Plant, Center of Biotechnology of Borj Cedria (CBCC), Hamman-Lif, Tunisia

**Keywords:** anthelmintic, herbal products, micropropagation, salt tolerant plants, saline agriculture, salt stress

Recruiting wild halophytes with economic potential was suggested several decades ago to reduce the problems associated with soil and water salinization and reduction of freshwater resources for agriculture. Most commercial crops are salt sensitive (glycophytes), while other crops (*e.g*. barley, oat and quinoa) are tolerant to moderate and high salinity. The growth of several halophytes is stimulated within a salinity range of 15 - 25 dS/m (8 - 15 g l^-1^) and, therefore, they are an important alternative to be cultivated in different saline systems, for sustainable water management and soil conservation, establishing cost-efficient and environmental-friendly agro-ecosystems and providing high added value products. Halophytes represent only 2% of terrestrial plant species but are present in about half the higher plant families and are valuable sources of bioactive molecules with multiple biotechnological applications (Ksouri et al., 2012). Such biochemical importance is linked to their habitat. Halophytes are the typical flora of saline environments, such as salt marshes, maritime dunes, and marine cliffs, where they are exposed to stressful abiotic conditions. Different criteria to classify halophytes (salt tolerant) and glycophytes are used. Halophytes are commonly defined as highly salt tolerant plants able to complete their life cycle under salinity conditions higher than 200 mM (Flowers and Colmer, 2008), or as those able to survive in high salinity soils, with a conductivity above 4 dS/m (Grigore et al., 2012). The high concentration of chloride and sodium ions causes osmotic stress, nutrient imbalance, ion toxicity, and oxidative stress, contributing to several deleterious effects on plants, such as stomatal closure, inhibition of photosynthesis and of cell division, and reduction in plant yield (Aslam et al., 2011; Acosta-Motos et al., 2017). To cope with such stress, halophytes are equipped with effective antioxidant mechanisms, including enzymatic and non-enzymatic tools, among which the synthesis of secondary metabolites, such as polyphenols and alkaloids (Ksouri et al., 2012) that, thanks to important biological properties, such as antioxidant, anti-inflammatory, and anti-parasitic, confer halophytes with important medicinal properties. Several species are used as medicinal and/or dietary plants, mainly in rural areas where traditional medicine is the only source of health treatments (Ksouri et al., 2012).

This Research Topic includes papers related with different aspects of halophytes, including adaptative molecular responses to salt stress, potential commercial uses, and propagation. The analysis of the salt-responsive proteome in plants allows understanding the intricate tools of plant salt tolerance, and until now, approximately 2100 salt-responsive proteins were described in different plant species, such as barley (Ras Rasoulnia et al., 2011), and sugar beet (Wang et al., 2019). Such proteins are involved in the regulation of different processes that may be variety-dependent, including photosynthesis and protein synthesis (Guo et al., 2012). In the first Research Topic paper, Chen et al. made a proteomic comparative analysis of the molecular mechanisms of tolerance to salt stress of a salt-tolerant (Vao-9) and a salt-sensitive (Bai5) oat (*Avena sativa* L.) cultivar. A total of 2631 proteins (2471 in Bai5 and 2493 in Vao-9), were qualitatively detected by mass spectrometry, and 138 were specific in Bai5 and 160 in Vao-9. The differentially expressed proteins (DEPs) were 76 in Bai5 and 214 in Vao-9, and in both cultivars the polypeptides up-regulated by salinity treatment (150 mM, NaCl:Na_2_SO_4_) were about twice the down-regulated ones. More proteins belonging to carbohydrate and energy metabolism, protein synthesis, and second metabolism were found in the salt-tolerant cv. Other functional categories of DEPs included photosynthesis and electron transport chain, signal sensing and transduction. These results provide an important basis for further research on the underlying mechanisms of salt tolerance in oats and other species.

Halophytes have ethnoveterinary uses, and can be exploited as sources of veterinary products, including antiparasitic agents (Oliveira et al., 2021a; Oliveira et al., 2021b). Oliveira et al. appraised the influence of environmental and phenological factors on the chemical and anthelmintic properties towards *Haemonchus contortus* and *Trichostrongylus colubriformis*) of *Cladium mariscus* L. Pohl (sawgrass), collected in the Southern Portugal. The anthelmintic activity was strongly influenced by season, with a higher activity being observed for samples collected in summer, and by anatomical organ, with inflorescences being more active. Polyphenols seem to be the main metabolites responsible for the egg hatching inhibitory properties, but not for the anti-larval effects. This study highlights sawgrass as a potential source of anthelminthic compounds to be used in veterinary.

Single-country endemic plants have a high importance as sources of high added value products (Sefi et al., 2021). Accordingly, Youssef et al. reported that *Limonium spathulatum* (Desf.) Kuntze leaves from Tunisian sea cliffs were good source of minerals and fibers, while ethanol and hydroethanol extracts exhibited high *ex vitro* antioxidant properties, and were rich in bioactive molecules, including hydroxybenzoic and hydroxycinnamic acids, and flavonoids. The authors suggested the possible use of extracts of *L. spathulatum* as herbal products to improve general health and well-being, and/or as food additives for food preservation. When developing medicinal plants as commercial crops, the production of enough number of plants, with desired biological properties, must be ensured. The use of plant tissue culture techniques (*e.g*., *in vitro* micropropagation) allows obtaining biomass with standardized contents of target bioactive metabolites from selected genetically identical plants (Moraes et al., 2021). In Custódio et al., a micropropagation protocol is established for *Polygonum maritimum* L. (sea knotgrass), a salt tolerant plant rich in bioactive flavonoids, including myricetin and quercetin glycosides. Combining 6-benzylaminopurine (BA, 3 mg/L) and indole-3-acetic acid (IAA, 0.1 mg/L) allowed for the upmost shoot formation, while rooting was improved by kinetin (KIN, 2 mg/L) and BA (3 mg/L) + IAA (0.1 mg/L). Plants derived from the control medium had the highest survival percentage in the acclimatization process. The authors suggest the optimized protocol as a mean to obtain biomass from sea knotgrass for the extraction of bioactive molecules.

Overall, the articles included in this Research Topic increased knowledge on different aspects of halophytes exploitation, which can be the basis for further research aiming the full and sustainable use of such important plants.

## Author contributions

LC wrote the first draft of the manuscript. All authors contributed to manuscript revision, read, and approved the submitted version.
